# Analysis of a Preimplantation Genetic Test for Aneuploidies in Embryos from Colombian Couples: A Report of Cases

**Published:** 2020

**Authors:** Diana Cárdenas-Nieto, Maribel Forero-Castro, Harold Moreno-Ortiz, Elkin Lucena-Quevedo, Juliana Cuzzi, Clara Esteban-Pérez

**Affiliations:** 1- Biological Sciences Department, Biomedical Sciences Research Group, Universidad Pedagógica y Tecnológica de Colombia, Tunja, Colombia; 2- Fertility and Sterility Colombian Center (CECOLFES), Bogotá, Colombia; 3- Genesis Genetics Brasil, Laboratory PGD, Sao Paulo, Brazil

**Keywords:** aCGH, Assisted reproduction techniques (ARTs), Copy number variations, Preimplantation genetic test for aneuploidies (PGT-A)

## Abstract

**Background:**

Assisted reproduction techniques (ARTs) and the preimplantation genetic test for aneuploidies (PGT-A) help couples with fertility problems to achieve a healthy live birth around the world. The aim of this study was to determine the rate of whole chromosomal copy number variations in embryos from couples undergoing ART and PGT-A, associations of chromosomal variations with embryo morphological parameters, and their relationship to maternal age.

**Methods:**

This study included a retrospective analysis of the number of whole chromosomal copies identified by aCGH in embryos from couples undergoing ART.

**Results:**

Seventy-six embryos from 29 couples using their own gametes were analyzed, of which 25 (32.9%) were chromosomally normal, and 51 (67.1%) were abnormal. Eleven embryos were evaluated from the group of couples with donated gametes, of which 5 (45.4%) embryos were chromosomally normal, and 6 (54.5%) embryos were abnormal. The main aneuploidies observed were trisomy X (7.8%), trisomy 21 (5.9%), trisomy 9 (3.9%), monosomy 11 (3.9%), monosomy 13 (3.9%) and monosomy X (3.9%), and the principal chromosomes affected were 19, X and 13. A significant association was found between the quality of the embryo and the genetic condition: embryos with euploidy and aneuploidy (p=0.046).

**Conclusion:**

The rate of aneuploidies from couples with their own gametes was 67.1% (51/76) and from couples with donated eggs and/or sperm was 54.5% (6/11). The quality of the embryo determinated by the morphological parameters was not associated with the embryo genetic status, and also there was no association between maternal age and aneuploidy rate.

## Introduction

Embryo development is a complex process that begins with oocyte fertilization by the spermatozoa developing a zygote and later on a blastocyst (1, 2). In some cases, this cell process results in an aneuploid embryo, which refers to an embryo with an unbalanced genome in which broad and/or focal losses and/or gains of chromatin can be present (3, 4). Aneuploidies may affect any of the 46 chromosomes affecting normal embryo development and the rate of embryo implantation, increasing the rate of spontaneous miscarriage and the rate of congenital defects (3, 5, 6).

Different factors, such as advanced maternal age, male factor, male factor infertility and recurrent miscarriages can affect the success of pregnancy and live births in couples around the world. Assisted reproductive technologies (ARTs) help infertile couples to achieve a pregnancy and often culminate in a live birth (7, 8). ARTs, including *in vitro* fertilization (IVF) and intracytoplasmic sperm injection (ICSI), have contributed to the birth of approximately 5 million children worldwide (9, 10).

The preimplantation genetic test for aneuploidies (PGT-A) has been used to identify genetically normal embryos, increasing the probability of a person achieving a healthy pregnancy and therefore a successful birth per embryo transfer (11). The implementation of the array-based analysis technique (aCGH) allows the screening of all 46 chromosomes and enables efficient identification of chromosomal abnormalities in comparison with techniques such as fluorescence in situ hybridization (FISH) (12, 13).

The achievement of ART depends on multiple factors, such as the genetic status of the embryo, quality of the embryo, endometrial receptivity, and embryo transfer techniques (14, 15). The rate of chromosomal abnormalities in human embryos is approximately 21% to 85%. This wide variation is related to maternal factors such as age, earlier miscarriages, previous congenital diseases, and recurrent implantation failures, which decrease the likelihood of a live birth (12, 14, 16–22). Although multiple factors may contribute to the success of ART, such as IVF/ICSI, the main factor influencing the treatment outcome is the ability to select a genetically competent embryo (17).

FISH was the first technique used in PGT-A. The FISH technique is a molecular cytogenetic method that permits the identification of a limited number of chromosomes by using simultaneously fluorescent labeled probes. The main probes used in the FISH technique are for the characterization of chromosomes 13, 18, 21, X, and Y, covering approximately 90–95% of aneuploidies in live-born infants (23). Nevertheless, the advances in ART and PGT-A have allowed comprehensive chromosome screening (CCS) of all 46 chromosomes. aCGH has been clinically implemented due to its advantages in identifying broad and/or focal losses and/or gains across the genome (24–26).

Previous studies have identified that PGT-A can increase the successful embryo implantation and pregnancy rates from couples with advanced maternal ages, history of recurrent abortion, recurrent implantation failure, and pregnancies with aneuploidies (5, 27, 28). In addition, PGT-A provides significant information from a single embryo prior to uterine transfer, improving the success rate (29). Therefore the aim of this study was to determine the rate of whole chromosomal copy number variations in embryos from couples undergoing ART, and their association with embryo morphological parameters, and maternal age.

## Methods

### Study design:

This study was a retrospective analysis of variations in whole chromosomal copy number identified in embryos from couples undergoing ART in Fertility and Sterility Colombian Center (CECOLFES-Bogotá, Colombia) from 2016 to 2018. The study was approved by the appropriate ethics review committee of CECOLFES.

All patients were counseled by fertility specialists regarding ART and PGT-A.

### Study objects:

A total of 87 embryos were included in the present study; there were 76 embryos from 29 couples without any gamete donation, and 11 embryos from 8 couples with donated gametes. Couples with advanced maternal age (≥35 years), male infertility, a history of recurrent miscarriage, recurrent implantation failure, and previous aneuploidies were included. All couples received complete genetic counseling concerning possible advantages, previously reported success rates, and risks of misdiagnosis with the use of PGT-A analysis by array-CGH. Signed consent forms were obtained from all included couples in the present study.

### IVF/ICSI:

Personalized ovarian stimulation protocols were performed according to ovarian reserve analysis for each patient, followed by oocyte retrieval. The MII oocytes were fertilized and cultured in groups and droplets of one-step human embryo culture medium at 37*°C*, with 6% CO_2_ and 5% O_2_. On day 5 or 6, blastocysts with differentiated inner-cell mass (ICM) and trophectoderm (TE) were biopsied.

### Embryo biopsy and PGT-A:

The blastocyst zone was opened at the side opposite to the inner cell mass. The Likos laser was used for biopsy, as reported previously (30). Laser pulses between two trophectoderm cells and mechanical separation were applied to isolate 3–10 trophectoderm cells. The biopsied cell samples were pipetted into individual PCR tubes previously labeled with the patient ID and the number of embryos. Sample tubes were frozen and shipped with ice packs and analyzed for PGT-A using Next Generation Sequencing (NGS) at either Genesis Genetics in Brazil, São Paulo or Houston TX. Both facilities used the VeriSeq platform (Illumina, San Diego, CA). Reports of euploid embryos were required to proceed for thawing of frozen embryos for elective frozen embryo transfer.

### Whole-genome amplification and DNA quantification:

Blastocyst biopsy was performed at the IVF laboratory of CECOLFES (Bogota, Colombia) according to international protocols. The biopsied cells were lysed to release and retrieve the genomic DNA. Subsequently, fragmented DNAs were preamplified and amplified according to the manufacturer’s instructions for the Sureplex WGA system (Sureplex, Illumina, USA). Briefly, the cells were lysed in Sureplex cell extraction buffer and cell extraction master mix and incubated at 75*°C* for 10 *min* followed by 95*°C* for 4 *min*. The DNA was randomly fragmented in Sureplex pre-amplification mix and incubated for 1 cycle at 95*°C* for 2 *min*, 12 cycles at 95*°C* for 15 *s*, 15*°C* for 50 *s*, 25*°C* for 40 *s*, 35*°C* for 30 *s*, 65*°C* for 40 *s*, and 1 cycle of 4*°C* infinite hold. Finally, the Sureplex amplification mix was added, and the final program was as follows: 14 cycles of 95*°C* for 15 *s*, 65*°C* for 1 *min*, 75*°C* for *min*, and holding at 4*°C*. The dsDNA High-Sensitivity (HS) Assay Kit (Qubit®, Life Technologies, USA) was used to quantify the concentration of amplified DNAs. The amplification products from 87 biopsied embryo cells were used for aCGH analysis, and the NGS approach was used as a validation test.

### aCGH analysis:

All amplified WGA products were assessed at Genesis Genetic Laboratory (Brazil, São Paulo) according to the protocols. All samples were tested on aCGH 24sure V3 microarray (Illumina, Inc.). The products and the reference DNA were labeled with Cy3 and Cy5 fluorophores using random primers for 2–4 *hr*. Slide preparation, hybridization, scanning and image analysis were performed according to the manufacturer’s instructions. Autosomal profiles were analyzed to identify whole chromosomal gain or loss ratios using a 3xSD assessment, greater than± 0.3log2 ratio call, or both. For quality control of hybridization, female samples were hybridized with known male references sample (Sex mismatch) with a consistent gain of chromosome X and consistent loss of chromosome Y. More details of the aCGH testing procedure can be found in Lai et al.’s study (31).

### NGS analysis:

Next, NGS analysis was used as a validation test in some embryos analyzed. Amplification products were processed, bar-coded, purified, pooled, denatured, and then sequenced to prepare DNA libraries following the manufacturer’s guidelines (VeriSeq PGT-A Illumina, Inc). The MiSeq Reagent Kit v.3 (Illumina, Inc.) was used on a MiSeq System (Illumina, Inc.). The generated bioinformatics data were analyzed by BlueFuse Multi Software (Illumina, Inc.). Embryos were identified by a median chromosomal copy number deviation from the default copy number. Possible trisomy or monosomy of embryo autosomal chromosomes was seen as copy numbers >2 or <2, respectively. Details of preparation procedures and the determination of automated copy number for each chromosome on BlueFuse Multi Software (Illumina, Inc.) were described in Fiorentino et al.’s (6) and Lai et al.’s study (31).

### Statistical analysis:

The data were presented as percentages, the parametric continuous data as averages with standard deviations, and nonparametric continuous data were presented as medians with maximum and minimum values. Comparisons of the percentage distribution between the groups were analyzed by the chi-square test. Significant differences were defined as a two-sided p-value<0.05. All the analyses were generated using the IBM software SPSS Statistics 22.0.

## Results

### Clinical characteristics of couples:

Table 1 shows the clinical characteristics of all included couples (n=37); 29/37 (78%) of couples used their own eggs and sperm, and 8/37 (22%) couples used donated eggs or sperm.

**Table 1.** Characteristics of couples included in the study

**Characteristics**	**Non-donor**	Donor^*^
All couples, n(%)	29 (78)	8 (22)
Maternal age in years, median (range)	41 (29–49)	42 (35–47)
29–34, n(%)	3 (10.3)	0 (0)
35–40, n(%)	9 (31)	2 (25)
41–46, n(%)	14 (48.3)	5 (62.5)
>46, n(%)	3 (10.3)	1 (12.5)
Advanced maternal age in years		
<35, n(%)	3 (10.3)	0 (0)
≥35, n(%)	26 (89.7)	8 (100)
Paternal age in years, median (range)	43 (25–59)	40 (36–53)
25–33, n(%)	2 (6.9)	0 (0)
34–39, n(%)	6 (20.7)	3 (42.8)
40–47, n(%)	14 (48.3)	2 (28.6)
> 48, n(%)	7 (24.1)	2 (28.6)
Advanced paternal age in years		
<40, n(%)	8 (27.6)	3 (42.8)
≥40, n(%)	21 (72.4)	4 (57.2)
Cytogenetics, n	10	2
Normal maternal karyotype, n(%)	9 (100)	1 (100)
Altered maternal karyotype, n(%)	0 (0)	0 (0)
Normal paternal karyotype, n(%)	9 (90)	1 (100)
Altered paternal karyotype,n(%)	1 (10)	0 (0)
Recurrent pregnancy loss, n(%)	4 (13.8)	0 (0)
Male factor, n(%)	17 (73.9)	4 (50)
Primary infertility, n(%)	8 (40)	1 (33.4)
Secondary infertility, n(%)	12 (60)	2 (66.7)
Previous ART failure, n(%)	8 (27.6)	4 (50)
Previous aneuploidy,n(%)	3 (10.3)	2 (25)
Monosomy 13 and 19, n(%)	1 (33.3)	0 (0)
Monosomy 8, n(%)	0 (0)	1 (50)
Trisomy 18, n(%)	1 (33.3)	0 (0)
Trisomy 21, n(%)	1 (33.3)	1 (50)
Type of ART		
ICSI, n(%)	21 (72.4)	6 (75)
ICSI/IVF, n(%)	4 (13.8)	1 (12.5)
IVF, n(%)	4 (13.8)	1 (12.5)
Number of total cycles ART, n (median, range/mean±SD)	64 (2, 1–6)	18 (2±1)
Number of total cycles IVF, n (median, range)	14 (0, 0–3)	4 (0, 0–3)
Number of total cycles ICSI, n (median, range/mean±SD)	50 (1, 0–6)	14 (2±1)
Number of total cycles failure, n (median, range)	15 (0, 0–2)	5 (0, 0–2)
Number of obtained embryo, n (mean±SD)	121 (4±2)	18 (3±2)
Number of arrested embryo, n (median, range)	45 (1, 0–5)	7 (0, 0–3)
Number of genetically analyzed embryo, n (median, range)	76 (2, 7–1)	11 (1, 1–6)
Number of donor eggs, (mean±SD)	NA	10 (2 ± 1)
Number of couples with donor sperm, n (%)	NA	2 (37.5)

Abbreviations: NA= Not Applicable, ART= Assisted Reproductive Technology, ICSI= Intracytoplasmic Sperm Injection, IVF= *In vitro* Fertilization, ICSI/IVF= Intracytoplasmic Sperm Injection-*in vitro* Fertilization.

* Couples with donated eggs or sperm

The group of couples without any gamete donation (n=29) had a median female age of 41 years (Range 29–49 years). Approximately, 48.3% (14/29) of the women were aged between 41 and 46 years, and 89.7% (26/29) had advanced maternal age (≥35 years). The median male age was 43 (Range 25–59 years), with 48.3% (14/29) of men distributed in the range of 40 to 47 years and 72.4% (21/29) presenting advanced paternal age (≥40 years). Ten blood karyotypes were performed on couples without any gamete donation identifying one abnormal karyotype (10%) with a Robert-sonian translocation 45,XY,rob (13;14)(q10;q10). Four couples presented recurrent pregnancy loss (13.8%), 17 couples showed male factors (73.9%), 8 (40%) and 12 (60%) couples showed primary and secondary infertility, 27.6% (8/29) of the couples had gone through failed assisted reproduction treatments and 10.3% (3/29) of couples presented pregnancies with previous aneuploidies: monosomy 13 and 19, trisomy 18 and trisomy 21 (Table 1).

A total of 64 cycles of ART (ICSI or IVF) were performed in the group of couples with their own gametes, with a median of 2 (Range 1–6) cycles per couple. A total of 72.4% of the couples in the group with their own eggs were treated with ICSI, 13.8% were submitted to conventional IVF, and 13.8% were treated with a combination of ICSI/IVF. A total of 121 embryos were obtained from 29 couples, and seventy-six embryos were genetically analyzed (Table 1).

In the group of couples with eggs and/or sperm donated for ART (n=8), the median female age was 42 years (Range 35–47 years), with the main percentage being between 41 and 46 years old (62.5%, 5/8), and the median male age was 40 years (Range 36–53 years). All female partners showed advanced maternal age (≥35 years), and 57.2% (4/7) of males had advanced paternal age. Any blood karyotypes were performed on this group. Four couples presented male factors and primary and secondary infertility was present in 1 and 2 cases, respectively. Fifty percent (4/8) of couples had previous unsuccessful assisted reproductive treatments. Two couples out of 8 (25%) presented two previous pregnancies with aneuploidies: monosomy 8 and trisomy 21 (Table 1). A total of 18 ART cycles were performed in the group of couples with gamete donation (Eggs and/or sperm), with a mean of 2±1 cycles per couple. A total of 9 eggs were donated to couples from healthy women with a mean of 2±1 per couple, and two couples received a sperm donation. Of these couples, 75% (6/8) were treated with ICSI, 12.5% (1/8) with IVF and 12.5% (1/8) with ICSI/VF. A total of 18 embryos from 8 couples were obtained, and 11 were genetically analyzed (Table 1).

### Embryo characteristics:

In this study, among the 121 embryos obtained by ART from 29 couples with own gamete, 76 were included in the genetic study. Table 2 presents the characteristics of the analyzed embryos: 71/76 (93.4%) embryos were obtained from females with advanced maternal age (>35 years), and 56/76 (73.7%) embryos were obtained from males with advanced paternal age (>40 years). Approximately, 58/76 (76.3%) embryos were obtained by ICSI, 14/76 (18.4%) embryos by IVF and 4/76 (5.3%) by a combination of IVF and ICSI.

**Table 2.** Characteristics of embryos analyzed in the study

**Characteristics**	**Non-donor**	Donor^*^
**All embryos, n(%)**	76 (87.3)	11 (12.7)
**Maternal age (years)**		
29–34, n(%)	5 (6.6)	0 (0)
35–40 years, n(%)	18 (23.7)	3 (27.3)
41–46 years, n(%)	47 (61.8)	6 (54.5)
>46 years, n(%)	6 (7.9)	2 (18.2)
**Advanced maternal age (years)**		
<35, n(%)	5 (6.6)	0 (0)
≥35, n(%)	71 (93.4)	11 (100)
**Paternal age(years)**		
25–33, n(%)	3 (3.9)	0 (0)
34–39, n(%)	17 (22.4)	4 (40)
40–47, n(%)	34 (44.7)	4 (40)
>48, n(%)	22 (28.9)	2 (20)
**Advanced paternal age (years)**		
<40, n(%)	20 (26.3)	4 (40)
≥40, n(%)	56 (73.7)	6 (60)
**Type of ART from which the embryo was obtained**		
ICSI, n(%)	58 (76.3)	10 (90.9)
ICSI/IVF, n(%)	4 (5.3)	0 (0)
IVF, n(%)	14 (18.4)	1 (9.1)
**Donor**		
Eggs, n(%)	NA	9 (81.8)
Sperm, n(%)	NA	2 (18.2)
Substitute mother, n(%)	5 (6.6)	0 (0)
**GENETIC STUDY**		
Number of cycle from embryo, median (range)	2 (1–5)	1
Gonadotropin dosage, median (range)	170 (75–225)	150
Duration of stimulation, median (range)	9 (8–10)	9
Day of biopsy, median (range)	6 (5–7)	6
**Genetic result by aCGH**		
Euploidy, n(%)	25 (32.9)	5 (45.5)
Aneuploidy, n(%)	51 (67.1)	6 (54.5)
Number of chromosomes affected, median (range)	2 (0–5)	2 (0–5)
**Destination of embryo**		
Transferred, n(%)	17 (22.4)	4 (36.4)
Vitrificated, n(%)	59 (77.6)	6 (54.5)
Investigation, n(%)	0 (0.0)	1 (9.1)
Implanted embryo, n(%)	15 (88.2)	2 (50)
Positive hCG, n(%)	8 (47.1)	0 (0)
Biochemical pregnancy, n(%)	5 (62.5)	0 (0)
Spontaneous Abortion, n(%)	0 (0)	0 (0)
Ongoing pregnancy, n(%)	1 (100)	0 (0)
Live birth, n(%)	2 (100)	0 (0)

Abbreviations: NA= Not Applicable, ART= Assisted Reproductive Technology, ICSI= Intracytoplasmic Sperm Injection, IVF= *in vitro* fertilization, ICSI/IVF= Intracytoplasmic sperm injection-*in vitro* fertilization.

* Couples with donated eggs or sperm

The embryos obtained from couples with their own gametes were genetically analyzed by aCGH (n=76). A total of 25 (32.9%) embryos showed normal results, and fifty-one (67.1%) embryos displayed chromosomal aneuploidy. At present, 77.6% of embryos (59/76) remain vitrified, and 22.4% of embryos (17/76) were transferred to the uterus. From the transferred embryos, 88.2% demonstrated successful implantation. Forty-seven percent of embryos presented positive hCG.

In the group of couples with gamete donation for ART, 11 embryos were analyzed: 9 (81.8%) with donated eggs and 2 (18.2%) with donated sperm. Ten (90.9%) embryos were obtained through the ICSI procedure, and only one (9.1%) embryo was obtained by IVF. All embryos were genetically analyzed by aCGH (n=11). A total of five (45.5%) embryos were euploid, and six (54.5%) embryos showed abnormal results (Table 2).

### Genetic analysis results:

The embryo aneuploidy rate was 67.1% (51/76) from couples with their own gamete, and the main aneuploidy was complex aneuploidy of 5 or more chromosomes, which was observed in 14/51 (27.5%) embryos. A total of 13/51 (25.5%) embryos showed a broad gain of entire chromosomes as follows: broad gain of one entire chromosome (11/13), two entire chromosomes (1/13), and three entire chromosomes (1/13). A total of 12/51 (23.5%) embryos showed broad loss of entire chromosomes as follows: broad loss of one entire chromosome (10/12), two entire chromosomes (1/12), and three entire chromosomes (1/12). A total of 6/51 (11.8%) embryos showed a broad loss or gain of entire chromosomes, 2/51 (3.9%) embryos showed a focal loss of chromosomes, 2/51 (3.9%) showed a focal gain of chromosomes, 1/51 (2%) showed a broad gain and focal loss of chromosomes, and 1 (2%) embryo showed a broad focal gain and focal loss of chromosomes (Supplementary table 1).

The main abnormalities observed in embryos with no complex aneuploidies were trisomy X (7.8%), trisomy 21 (5.9%), trisomy 9 (3.9%), monosomy 11 (3.9%), monosomy 13 (3.9%) and monosomy X (3.9%). Other abnormalities were present in 2% of all aneuploidies (Supplementary table 1).

**Figure 1.**
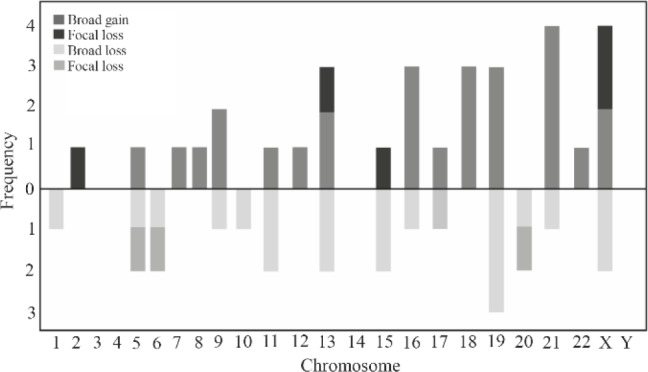
The frequency of broad and/or focal gain and/or loss of DNA in each chromosome. Chromosomes 3, 4, 14 and Y did not show a gain or loss of DNA, chromosomes 2, 7, 8, 12, 18 and X only showed a gain in the genetic material, and chromosomes 1, 10 and 20 showed broad and/or focal loss of genetic material. The other chromosomes showed broad and/or focal gains and losses

No aneuploidies were observed in the 3, 4, 14 and Y chromosomes. Broad and/or focal gain and/or loss of chromosomes was observed in the X chromosome with two broad gains, two focal gains and two broad losses. Chromosome 21 showed four broad gains and one broad loss. Chromosome 19 showed three broad losses and three broad gains. Chromosome 13 presented two broad losses and one focal gain. Broad and focal loss was observed on chromosomes 1, 6, 10, and chromosome 20 showed a broad and/or focal loss. Broad and focal gain was observed in chromosome 2 with a focal gain, and chromosomes 7, 8, 12 and 22 only showed a broad gain. Chromosomes 5, 9, 11, 15, 16 and 17 showed broad and/or focal gain and/or loss (Figure 1).

In the group of couples with eggs and/or sperm donated for ART, a total of 6 (54.4%) embryos presented abnormal results; one broad loss in chromosome 20, one broad gain in the chromo- some 21, one broad gain and loss involving chromosomes 18,19 and 20, and three complex aneuploidies affecting 5 or more chromosomes (Supplementary table 1).

### Association of maternal age and embryo quality:

Supplementary table 2 shows the clinical characteristics corresponding to embryo quality from 76 couples without any gamete donation. The high- quality embryos from these couples represent those with a Gardner blastocyst grading scale score equal to or greater than 3BB; indeed, the total high-quality embryo formation rate was 89.3% (68/76). Among these embryos, 27.6% (21/76) had a quality of 4BB, 21.1% (16/76) had a quality of 4AA, and 17.1% (13/76) of embryos had a quality corresponding to 4AB. The percentage of high-quality embryos was not significantly correlated with maternal age (p=0.329). Only 10.6% (8/76) of embryos had a quality below 3BB, which came from a woman with advanced maternal age (≥35 years). All embryos from women aged between 29 and 34 years had a high quality score (4AB, 4BB, 5AA).

### Association of male factors and embryo quality:

Forty-two embryos obtained from couples with their own gametes had male factor data. Embryo formation rates from couples in this group were as follows: 17.6% (9/42) of embryos were obtained from sperm samples with normozoospermia, 37.3% (19/42) of embryos were obtained from sperm samples with asthenoteratozoospermia, 9.8% (5/42) were from sperm samples with cryptozoospermia, 7.8% (4/42) of embryos were from sperm samples with oligoasthenoteratozoospermia, and 27.5% (14/42) were from sperm samples with teratozoospermia. In the present study, no association was found between male factors, embryo genetic status (p=0.275), and the implantation rate (p=0.435).

### Association of quality of embryo and euploidy/aneuploidy status:

Supplementary table 3 shows the genetic conditions of the embryos analyzed in the present study. Although there was no difference between the frequency of aneuploidies in embryos from couples with donated gametes and couples with their own gametes (54.4% *vs*. 67.1%, p= 0.421), there were differences among the aneuploidies observed in the embryos from couples without any gamete donation (p=0.011). In 3AA category embryos, no aneuploidies were observed (0% *vs*. 5.3%, p=0.017).

It should be noted that, in the group of embryos from couples with their own gametes, although there were no significant differences, the highest frequency of aneuploidies was observed in embryos with 4BB quality (22.4% *vs*. 5.3%, p= 0.189) and 4AA (14.5% *vs*. 7.9%, p=0.887), and the majority of euploidies were observed in embryos with 4AA (6/25), 4AB (5/25) and 4BB (4/25) quality. In the case of the group with gamete donation, the quality of the embryo was not significantly associated with the genetic condition of the embryo.

The quality of the embryo was not associated with the embryo genetic status: normal, broad and/or focal loss and/or gain (p=0.999). Nevertheless, 85.7% of embryos with low quality (Below 3BB on the Gardner blastocyst grading scale) showed normal genetic results, and 27.9% of embryos with high quality had normal genetic results (Supplementary table 4).

### Association of the day of biopsy and embryonic euploidy/aneuploidy status:

Regarding the association between the day of embryo biopsy and embryo genetic status, no significant correlation was found in the group of embryos from couples with their own gametes (p=0.208). A total of 28 embryos were analyzed on day 5; 60.7% (17/28) showed an abnormal result, and 39.3% (11/28) were euploid. A total of 61.8% (47/76) of embryos were analyzed on day 6; 72.4% (34/47) showed aneuploidies, and 27.6% (13/47) had normal results. Only one embryo was biopsied on day 7 and showed a normal result.

### Association of whole-chromosome abnormalities with maternal age:

Supplementary table 5 depicts the observed chromosomal abnormalities according to maternal age. Twenty-five embryos (32.9%) of all analyzable embryos were euploid, and fifty-one embryos (67.1%) were aneuploid. Twenty-three embryos (30.3%) had one affected chromosome, seven embryos (9.2%) showed two abnormal chromosomes, 5 embryos (6.6%) presented three affected chromosomes, 2 embryos (2.6%) showed four affected chromosomes and fourteen embryos (18.4%) had five or more affected chromosomes. The euploidy/aneuploidy status (p= 0.209), the number of affected chromosomes (p= 0.757) and the class of the chromosome abnormality (p=0.293) were correlated with maternal age in the group of couples with any type of donation.

The embryos from women between the ages of 29 and 34 years showed aneuploidies that affected one chromosome or five or more chromosomes, and the embryos from women between 35 and 40 years presented aneuploidies in one, two, three and five or more chromosomes. In women between 41 and 46 years old, the embryos had aneuploidies that could affect one to five or more chromosomes, and the embryos from women between 41 and 46 only showed aneuploidies in one or two chromosomes.

The main class of chromosomal abnormalities in embryos from women between 29 and 34 years of age was characterized as complex abnormalities, where 5 or more chromosomes were compromised. In embryos from women between 35 and 40 years old, the main observed aneuploidy was a broad gain. In embryos from women between 41 and 46 years old, the main classes of chromosomal abnormalities were complex abnormalities followed by broad loss and broad gain of chromosomes. Finally, embryos from women older than 46 years of age were classified as having a broad loss, both broad and focal losses and both broad gains and losses (Supplementary table 6).

### Cycle outcome:

From the total number of transferred embryos, 58.8% (10/17) of embryos were from women between 41 and 46 years of age. In this group, 60% (6/10) of embryos were transferred into the uterus, and 33.3% (2/6) of embryos showed positive hCG but did not show ultrasound pregnancy signs. The 35.3% (6/17) of transferred embryos were from women between 35 and 40 years of age. In this group, all embryos exhibited successful implantation, one was a biochemical pregnancy and only one embryo succeeded in a live birth. The 5.9% (1/17) of transferred embryos were from women with over 46 years of age, and the embryo exhibited successful implantation and ongoing pregnancy took place. The rate of transferred embryos (p=0.186), implanted embryos (p= 0.452), positive hCG results (p=0.446) and biochemical pregnancy (p=0.293) were not associated with maternal age in the group of couples with their own gametes (Supplementary table 7).

## Discussion

PGT-A is performed to increase the rate of a healthy pregnancy and genetically normal live births in couples receiving ART. The PGT-A technique is recommended for couples with a history of recurrent implantation failure, recurrent miscarriages, advanced maternal age, and male infertility factors (32). Chromosomal abnormalities might originate from controlled ovarian hyperstimulation during IVF/ICSI cycles and are a consequence of low oocyte and embryo quality. A negative effect of the fertilization of low-quality oocytes is the high rate of errors in meiotic divisions that could lead to an increase in embryo development arrest, embryo implantation failures, miscarriage rates, and a high risk of complications during pregnancy (33–37).

In the present study, aCGH technology was used to analyze all embryo chromosomes more and efficiently identify chromosomal abnormalities compared to fluorescence in situ hybridization in PGT-A (38–41). Despite embryonic development and chromosomal stability directly affecting embryo development and implantation, array-based analysis allows the overview of all chromosomes from a small number of cells from the embryo to determine their genetic status (39). The use of these technologies has improved the success rate of healthy live births and the detection of chromosomal broad losses and/or gains, and chromosomal focal losses and/or gains in the embryos before embryo transfer (12).

In our study, 29 couples with their own gametes had aneuploidy in 51 analyzed embryos (67.1%), and 15 couples with their own gametes after PGT-A had euploidy in 25 analyzed embryos (32.9%). Some authors concluded that in embryos from women with advanced maternal age, PGT-A improves clinical outcomes by successful embryo implantation; however, recurrent implantation failure is common due to de novo anomalies or unknown paternal factors (39, 42). Since the implementation of PGT-A, scientists expected the rates of embryo implantation and pregnancy by euploid embryo transfer to increase; however, in our study of 17 transferred euploid embryos, two resulted in a live birth, and one ongoing pregnancy was reported with heart rate and screening for aneuploidies by hormones and anatomic fetal ultrasound at 12 weeks of pregnancy, indicating that there are many other factors that may affect clinical outcomes.

Our data demonstrate a significant association between the quality of the embryo and the genetic condition; euploid and aneuploid (p=0.046) embryos came from couples without any gamete donation. This finding is in agreement with Majumdar et al.’s (43) who demonstrate an association between the blastocyst morphology and the euploidy rate. However, these results differ from some published studies by Bazgar et al. (38) and Fesahat et al. (44), suggesting that the morphological characteristics of embryos obtained through ART (IVF/ICSI) are not completely consistent with the genetic result (Normal or abnormal). Alfarawati et al. (45) compared embryo morphology and rate of aneuploidy, found a weak correlation between variables and concluded that establishing the quality of an embryo does not ensure the euploidy of the embryo. According to our findings, the morphological parameters play an important role but do not always predict euploid embryos.

Regarding the rate of embryo aneuploidies identified on day 5 or 6, no significant association was found. However, an increase in the rate of aneuploid embryos on day 6 (34/51, 66.7%) compared to the rate on day 5 (17/51, 33.3%) was observed, possibly induced by the longer exposure to the *in vitro* conditions and hyperstimulation protocols in IVF/ICSI treatments. This finding is in agreement with Bazgar et al. (38) who found a higher rate of abnormal cells on day 7 compared with the related value on day 6.

It is well known that the embryo aneuploidy rate increases with a woman’s age, supporting previous reports (19, 36, 42, 43). However, in our study, no significant association was found between maternal age and chromosomal abnormalities, but there were only 3 patients younger than 35, so it is necessary to increase the population to establish an adequately powered association. Cases of broad chromosomal loss (8/12, 66.7%) and chromosomal gain (8/13, 61.5%) present mainly in women between 41 and 46 years of age were described in this study. These results confirm the observations by Franasiak et al. (46) regarding the high rates of monosomies and trisomies in women older than 40 years. In our study, the quality of the embryo was not related to maternal age, and this observation was comparable with Eaton et al. (47), who concluded that maternal age did not impact embryo morphology or the association with genetic status.

In a recent study, Ubaldi et al. (48) found that transfer of a euploid embryo in women ≥44 years of age resulted in a live-birth rate of >50%. In the present study, four embryos were transferred to women ≥44 years of age, and only one embryo transfer displayed biochemical pregnancy. This finding indicated the very low success of a live birth pregnancy in patients between 45 and 46 years of age.

In our study, the overall aneuploidy rate of analyzed embryos exceeded 50% (67.1%, 51/76) of the total embryos from mothers aged between 29 and 49 years. Our observations support findings by Capalbo et al. (49), who showed an aneuploidy rate of 55.5% (531/956) in embryos from couples with a maternal age between 26 and 44 years. Our findings are similar to those described by Ubaldi et al. (48), who observed an aneuploidy rate of 88.2% (165/187) in embryos from women between 44 and 46 years of age. Fesahat et al. (44) found a rate of chromosomal abnormalities of 62.9% (36/96) in embryos from women less than 35 years of age. In our study, no association between maternal age and aneuploidy rate was found; however, the aneuploidy rate decreased by approximately 10.8% in women with maternal ages less than 43 years (63.3%, 31/49) compared to women with maternal ages greater than 43 years (74.1%, 20/27).

Our study established no association between male factors, the genetic status of embryos (p= 0.275), and the embryo implantation rate (p= 0.449) in the couples with gamete donation. These results support the study of Mazzilli et al. (50), who showed no significant association between male factors, embryo genetic condition, and embryo implantation rate in 1,219 cycles performed on 1,090 couples. Additionally, Mazzilli et al. (50) concluded that the euploidy rate and implantation potential of embryos obtained through ICSI treatments are independent of sperm quality.

Our study shows that the embryo implantation rate of euploid embryos was 76.5% (13/17) whereas the global implantation rate of euploid embryos is close to 50%. Two different reasons explain embryo implantation failure, mainly endometrial receptivity and embryo quality (5). Higher quality blastocysts are a key factor in successful embryo implantation in assisted reproduction treatments. It was found that all successfully implanted embryos showed a high-quality grade, confirming observations by Su et al. (5), who showed that in 15 embryos undergoing PGT-A, only two embryos with a good quality grade exhibited successful implantation, supporting the idea of no correlation between morphology, ploidy status, and implantation.

Regarding chromosome abnormalities, in the current study, 50% (22/44) of aneuploidies involved a broad and/or focal loss, and the remaining 50% (22/44) involved a broad and/or focal-gain, 84.4% (33/39) of aneuploidies affected entire chromosomes, and 15.4% (6/39) only affected focal regions. Among 297 aneuploid embryos, Fragouli et al. (21) identified that 52% (652/1262) involved losses and 48% (610/1262) involved gains; the vast majority (94%, 1188/1262) affected entire chromosomes, and only 6% (74/1262) were segmental imbalances. The main affected chromosomes were 13, 19, 21, and X. Our results are similar to those of Capalbo et al. (49), who demonstrated that in 531/956 (55.5%) embryos analyzed, the majority of anomalies occurred in chromosomes 13, 15, 16, 21 and 22. Likewise, our findings agree with Franasiak et al. (46), who identified the main prevalence of chromosomal abnormalities in chromosomes 13, 15, 16, 18, 19, 21 and 22, concluding that the highest aneuploidy rates are related to the small structure of chromosomes. In a study performed by Soler et al. (51), trisomy 13 (6.5%, 31/980), 21 (12.2%, 55/980) and monosomy X (14.21%, 67/980) were very common in miscarriages, indicating that these abnormalities could cause spontaneous abortions.

To the best of our knowledge, this is the first study from our population; however, our results are comparable with other global reports. As a perspective, it is possible to increase the number of cases of PGT-A reported and evaluate the recent discussions regarding aneuploidy rescue and the presence of mosaicism. Some authors have reported the presence of a low level of mosaicism in natural and ART pregnancies due to the role of aneuploidies on entire chromosomes or focal deletions as a predictor of cell fate decisions between TE and ICM cells, providing important clues on the origin and evolution of embryonic mosaicism and the chances of successful pregnancy and live birth (33, 49, 52).

To our knowledge, this is the first study from our population, although our results are comparable with those of other global reports. For future studies, it is suggested to increase the sample size or more data could be collected from a multicentric perspective to establish a more associative relation between the parameters studied.

## Conclusion

In the present study, the rate of aneuploidies from couples with their own gametes was 67.1% (51/76) and from couples with donated eggs and/or sperm was 54.5% (6/11). The quality of the embryo determinated by the morphological parameters was not associated with the embryo genetic status, and also there was no association between maternal age and aneuploidy rate. This study supports the use of aCGH technology to improve ART success, and this method brings an overview of whole genome and allows identifying genetic copy number alterations in ART-derived embryos.

## Supplementary

**Supplementary table 1.** Whole chromosome abnormalities observed in all the embryos analyzed

**Class of chromosome abnormality**	**Chromosomes involved**	**Non-donor**	**Donor^*^**
Euploidy, n(%)	NA	25 (32.9)	5 (45.5)
Aneuploidy, n(%)	NA	51 (67.1)	6 (54.5)
Broad loss, n(%)						
	−11	2 (3.9)	0 (0)
	−13	2 (3.9)	0 (0)
	−16	1 (2)	0 (0)
	−18	1 (2)	0 (0)
	−19	1 (2)	0 (0)
	−20	0 (0)	1 (16.7)
	−21	1 (2)	0 (0)
	−X	2 (3.9)	0 (0)
	−18, −19	1 (2)	0 (0)
	−5, −12, −15, −19	1 (2)	0 (0)
Focal loss, n(%)			
	del(17)(q) del(5)(p14.1-pter)	1 (2)	0 (0)
		1 (2)	0 (0)
Broad and focal loss, n(%)			
	−X,del(6)(q)	1 (2)	0 (0)
Broad gain, n(%)			
	+7	1 (2)	0 (0)
	+9	2 (3.9)	0 (0)
	+13	1 (2)	0 (0)
	+21	3 (5.9)	1 (16.7)
	+X	4 (7.8)	0 (0)
	+13,+19	1 (2)	0 (0)
	+11,+21,+22	1 (2)	0 (0)
Focal gain, n(%)			
	dup(2)(p24.1-pter), dup(15)(q25.3-qter),dup(X)(q25-qter)dup(X)(pter-p22.2)	1 (2)	0 (0)
		1 (2)	0 (0)
Broad gain and loss, n(%)						
	+5, −15	1 (2)	0 (0)
	−9,+16	1 (2)	0 (0)
	−10,+17	1 (2)	0 (0)
	+16, −18	1 (2)	0 (0)
	+19, −20	1 (2)	0 (0)
	−18,+19, −20	0 (0)	1 (16.7)
	−1, −6,+8,+16	1 (2)	0 (0)
Focal and broad gain and focal loss,n(%)			
	dup(13)(q14.3–q31.3).+19.del(20)(p)	1 (2)	0 (0)
Complex Aneuploidy^†^, n(%)		14 (27.5)	3 (50)

Abbreviations: NA= not applicable

a: Couples with eggs or sperm donated

†: 5 or more chromosomes affected

**Supplementary table 2.** Quality of analyzed embryos obtained according to maternal age

**Age (years)**	**Total**	**1BC, n(%)**	**1CC, n(%)**	**2AB, n(%)**	**3AA, n(%)**	**3AB, n(%)**	**3BB, n(%)**	**4AA, n(%)**	**4AB, n(%)**	**4AC, n(%)**	**4BA, n(%)**	**4BB, n(%)**	**4BC, n(%)**	**4CC, n(%)**	**4CD, n(%)**	**5AA, n(%)**	**5AB, n(%)**	**5BB, n(%)**	**6AA, n(%)**
**Donor**	11 (100)	0 (0)	0 (0)	0 (0)	0 (0)	0 (0)	0 (0)	3 (27.3)	5 (45.5)	0 (0)	0 (0)	2 (18.2)	0 (0)	0 (0)	0 (0)	0 (0)	1 (9.1)	0 (0)	0 (0)
**29–34**	5 (6.6)	0 (0)	0 (0)	0 (0)	0 (0)	0 (0)	0 (0)	0 (0)	1 (1.3)	0 (0)	0 (0)	3 (3.9)	0 (0)	0 (0)	0 (0)	1 (1.3)	0 (0)	0 (0)	0 (0)
**35–40**	18 (23.7)	1 (1.3)	1 (1.3)	0 (0)	0 (0)	0 (0)	0 (0)	2 (2.6)	4 (5.3)	1 (1.3)	0 (0)	8 (10.5)	0 (0)	0 (0)	0 (0)	1 (1.3)	0 (0)	0 (0)	0 (0)
**41–46**	47 (61.8)	0 (0)	0 (0)	1 (1.3)	4 (5.3)	1 (1.3)	1 (1.3)	11 (14.5)	8 (10.5)	0 (0)	1 (1.3)	10 (13.2)	0 (0)	1 (1.3)	1 (1.3)	5 (6.6)	0 (0)	2 (2.6)	1 (1.3)
**>46**	6 (7.9)	0 (0)	0 (0)	0 (0)	0 (0)	0 (0)	0 (0)	3 (3.9)	0 (0)	0 (0)	1 (1.3)	0 (0)	1 (1.3)	0 (0)	1 (1.3)	0 (0)	0 (0)	0 (0)	0 (0)
**Total**	76 (100)	1 (1.3)	1 (1.3)	1 (1.3)	4 (5.3)	1 (1.3)	1 (1.3)	16 (21.1)	13 (17.1)	1 (1.3)	2 (2.6)	21 (27.6)	1 (1.3)	1 (1.3)	2 (2.6)	7 (9.2)	0 (0)	2 (2.6)	1 (1.3)

**Supplementary table 3.** Association of quality of embryo with euploidy/aneuploidy tatus

**Quality of embryo**	**Euploidy, n(%)**	**Aneuploidy, n(%)**	**p-value**
**Donor^*^**			
4AA	3 (27.3)	0 (0)	0.122
4AB	1 (9.1)	4 (36.4)	0.347
4BB	1 (9.1)	1 (9.1)	1.000
5AB	0 (0)	1 (9.1)	1.000
TOTAL	5 (45.5)	6 (54.4)	0.061
**No-Donor^†^**			
1BC	1 (1.3)	0 (0)	0.714
1CC	1 (1.3)	0 (0)	0.714
2AB	0 (0)	1 (1.3)	1.000
3AA	4 (5.3)	0 (0)	0.017
3AB	0 (0)	1 (1.3)	1.000
3BB	1 (1.3)	0 (0)	0.714
4AA	6 (7.9)	11 (14.5)	0.887
4AB	5 (6.6)	7 (9.2)	0.885
4AC	1 (1.3)	0 (0)	0.714
4BA	0 (0)	2 (2.6)	0.810
4BB	4 (5.3)	17 (22.4)	0.189
4BC	1 (1.3)	0 (0)	0.714
4CC	0 (0)	1 (1.3)	1.000
4CD	0 (0)	2 (2.6)	0.810
5AA	1 (1.3)	6 (7.9)	0.498
5BB	0 (0)	2 (2.6)	0.810
6AA	0 (0)	1 (1.3)	1.000
Total	25 (32.9)	51 (67.1)	0.011

* Embryos with eggs or sperm donated

† Embryos with any donation

Probabilities highlighted in bold indicate statistically significant results (p<0.05)

**Supplementary table 4.** Embryo quality and genetic condition of the embryos analyzed

**Genetic condition**	**Total**	**1BC, n(%)**	**1CC, n(%)**	**2AB, n(%)**	**3AA, n(%)**	**3AB, n(%)**	**3BB, n(%)**	**4AA, n(%)**	**4AB, n(%)**	**4AC, n(%)**	**4BA, n(%)**	**4BB, n(%)**	**4BC, n(%)**	**4CC, n(%)**	**4CD, n(%)**	**5AA, n(%)**	**5AB, n(%)**	**5BB, n(%)**	**6AA, n(%)**
**Donor**	11 (100)	0 (0)	0 (0)	0 (0)	0 (0)	0 (0)	0 (0)	3 (27.3)	5 (45.5)	0 (0)	0 (0)	2 (18.2)	0 (0)	0 (0)	0 (0)	0 (0)	1 (9.1)	0 (0)	0 (0)
**Normal**	25 (32.9)	1 (1.3)	1 (1.3)	0 (0)	4 (5.3)	0 (0)	1 (1.3)	6 (7.9)	5 (6.6)	1 (1.3)	0 (0)	4 (5.3)	1 (1.3)	0 (0)	0 (0)	1 (1.3)	0 (0)	0 (0)	0 (0)
**Broad loss**	13 (17.1)	0 (0)	0 (0)	1 (1.3)	0 (0)	1 (1.3)	0 (0)	2 (2.6)	1 (1.3)	0 (0)	1 (1.3)	3 (3.9)	0 (0)	0 (0)	0 (0)	3 (3.9)	0 (0)	1 (1.3)	0 (0)
**Focal loss**	1 (1.3)	0 (0)	0 (0)	0 (0)	0 (0)	0 (0)	0 (0)	0 (0)	0 (0)	0 (0)	0 (0)	1 (1.3)	0 (0)	0 (0)	0 (0)	0 (0)	0 (0)	0 (0)	0 (0)
**Broad and focal los**	3 (3.9)	0 (0)	0 (0)	0 (0)	0 (0)	0 (0)	0 (0)	1 (1.3)	0 (0)	0 (0)	0 (0)	0 (0)	0 (0)	0 (0)	0 (0)	0 (0)	0 (0)	0 (0)	0 (0)
**Broad gain**	13 (17.1)	0 (0)	0 (0)	0 (0)	0 (0)	0 (0)	0 (0)	1 (1.3)	3 (3.9)	0 (0)	1 (1.3)	6 (7.9)	0 (0)	0 (0)	0 (0)	1 (1.3)	0 (0)	1 (1.3)	0 (0)
**Focal gain**	2 (2.6)	0 (0)	0 (0)	0 (0)	0 (0)	0 (0)	0 (0)	1 (1.3)	0 (0)	0 (0)	0 (0)	0 (0)	0 (0)	0 (0)	0 (0)	1 (1.3)	0 (0)	0 (0)	0 (0)
**Broad gain and loss**	6 (7.9)	0 (0)	0 (0)	0 (0)	0 (0)	0 (0)	0 (0)	2 (2.6)	1 (1.3)	0 (0)	0 (0)	1 (1.3)	0 (0)	0 (0)	1 (1.3)	0 (0)	0 (0)	0 (0)	1 (1.3)
**Focal and broad gain and focal loss**	1 (1.3)	0 (0)	0 (0)	0 (0)	0 (0)	0 (0)	0 (0)	0 (0)	0 (0)	0 (0)	0 (0)	1 (1.3)	0 (0)	0 (0)	0 (0)	0 (0)	0 (0)	0 (0)	0 (0)
**Complex**	14 (18.4)	0 (0)	0 (0)	0 (0)	0 (0)	0 (0)	0 (0)	3 (3.9)	3 (3.9)	0 (0)	0 (0)	5 (6.6)	0 (0)	1 (1.3)	1 (1.3)	1 (1.3)	0 (0)	0 (0)	0 (0)
**Total**	76 (100)	1 (1.3)	1 (1.3)	1 (1.3)	4 (5.3)	1 (1.3)	1 (1.3)	16 (21.1)	13 (17.1)	1 (1.3)	2 (2.6)	21 (27.6)	1 (1.3)	1 (1.3)	2 (2.6)	7 (9.2)	0 (0)	2 (2.6)	1 (1.3)

**Supplementary table 5.** Chromosomal abnormalities according to maternal age

**Maternal age (years)**	**Number of total cycles TRA**	**Number of embryos obtained**	**Number of embryos arrested**	**Number of embryos analyzed (n,%)**	**Euploidy (n,%)**	**Aneuploidy (n,%)**	**No affected chromosome (n,%)**	**One affected chromosome (n,%)**	**Two affected chromosome (n,%)**	**Three affected chromosomes (n,%)**	**Four affected chromosomes (n,%)**	**Complex (more than 5 affected chromosome) (n,%)**
**Donor**	18	23	6	12 (100)	5 (45.5)	6 (54.5)	5 (45.5)	2 (18.2)	0 (0)	1 (9.1)	0 (0)	3 (27.3)
**29–34**	6	13	7	6 (7.3)	0 (0)	5 (6.6)	0 (0)	3 (3.9)	0 (0)	0 (0)	0 (0)	2 (2.6)
**35–40**	17	30	7	21 (25.6)	8 (10.5)	10 (13.2)	8 (20.5)	5 (6.6)	2 (2.6)	1 (1.3)	0 (0)	2 (2.6)
**41–46**	38	62	19	48 (58.5)	14 (18.4)	33 (43.4)	14 (18.4)	13 (17.1)	4 (5.3)	4 (5.3)	2 (2.6)	10 (13.2)
**>46**	3	14	6	7 (8.5)	3 (3.9)	3 (3.9)	3 (3.9)	2 (2.6)	1 (1.3)	0 (0)	0 (0)	0 (0)
**Total**	64	119	39	82 (100)	25 (32.9)	51 (67.1)	25 (32.9)	23 (30.3)	7 (9.2)	5 (6.6)	2 (2.6)	14 (18.4)

**Supplementary table 6.** Class of genetic condition to regard with maternal age

**Maternal age (Years)**	**Euploidy, n(%)**	**Broad Loss, n(%)**	**Focal Loss, n(%)**	**Broad and Focal Loss, n(%)**	**Broad Gain, n(%)**	**Focal Gain, n(%)**	**Broad gain and loss, n(%)**	**Broad and focal gainand focal loss, n(%)**	**Complex**
**Donor**	5 (45.5)	1 (9.1)	0 (0)	0 (0)	1 (9.1)	0 (0)	1 (9.1)	0 (0)	3 (27.3)
**29–34**	0 (0)	1 (1.3)	1 (1.3)	0 (0)	1 (1.3)	0 (0)	0 (0)	0 (0)	2 (2.6)
**35–40**	8 (10.5)	2 (2.6)	1 (1.3)	0 (0)	4 (5.3)	0 (0)	1 (1.3)	0 (0)	2 (2.6)
**41–46**	14 (18.4)	8 (10.5)	0 (0)	0 (0)	8 (10.5)	2 (2.6)	4 (5.3)	1 (1.3)	10 (13.2)
**>46**	3 (3.9)	1 (1.3)	0 (0)	1 (1.3)	0 (0)	0 (0)	1 (1.3)	0 (0)	0 (0)
**Total**	25 (32.9)	12 (15.8)	2 (2.6)	1 (1.3)	13 (17.1)	2 (2.6)	6 (7.9)	1 (1.3)	14 (18.4)

**Supplementary table 7.** Cycle outcome

**Maternal age (years)**	**Transferred Embryo (n,%)**	**Implanted Embryo (n,%)**	**Positive hCG (n,%)**	**Biochemical pregnancy (n,%)**	**Spontaneous abortion (n,%)**	**Ongoing pregnancy (n,%)**	**Live Birth (n,%)**
**Donor**	4 (100)	2 (50)	0 (0)	0 (0)	0 (0)	0 (0)	0 (0)
**29–34**	0 (0)	0 (0)	0 (0)	0 (0)	0 (0)	0 (0)	0 (0)
**35–40**	6 (35.3)	6 (40)	2 (33.3)	1 (20)	0 (0)	0 (0)	1 (50)
**41–46**	10 (58.8)	8 (53.3)	5 (83.3)	4 (80)	0 (0)	0 (0)	1 (50)
**>46**	1 (5.9)	1 (6.66)	1 (16.6)	0 (0)	0 (0)	1 (100)	0 (0)
**Total**	17 (100)	15 (88.2)	6 (40)	5 (83.3)	0 (0)	1 (20)	2 (40)
